# Rapid Determination of the Chemical Oxygen Demand of Water Using a Thermal Biosensor

**DOI:** 10.3390/s140609949

**Published:** 2014-06-06

**Authors:** Na Yao, Jinqi Wang, Yikai Zhou

**Affiliations:** Institute of Environmental Medicine, Tongji Medical College, Huazhong University of Science and Technology, 430030 Wuhan, China; E-Mails: 59040368yaona@sina.cn (N.Y.); victory1219fly@163.com (J.W.)

**Keywords:** chemical oxygen demand (COD), thermal biosensor, flow injection analysis system, periodic acid

## Abstract

In this paper we describe a thermal biosensor with a flow injection analysis system for the determination of the chemical oxygen demand (COD) of water samples. Glucose solutions of different concentrations and actual water samples were tested, and their COD values were determined by measuring the heat generated when the samples passed through a column containing periodic acid. The biosensor exhibited a large linear range (5 to 3000 mg/L) and a low detection limit (1.84 mg/L). It could tolerate the presence of chloride ions in concentrations of 0.015 M without requiring a masking agent. The sensor was successfully used for detecting the COD values of actual samples. The COD values of water samples from various sources were correlated with those obtained by the standard dichromate method; the linear regression coefficient was found to be 0.996. The sensor is environmentally friendly, economical, and highly stable, and exhibits good reproducibility and accuracy. In addition, its response time is short, and there is no danger of hazardous emissions or external contamination. Finally, the samples to be tested do not have to be pretreated. These results suggest that the biosensor is suitable for the continuous monitoring of the COD values of actual wastewater samples.

## 1. Introduction

In recent years, increasing attention has been devoted to the removal of organic contaminants from wastewater. With water pollution becoming a serious issue, water quality assessment and pollutant control have become global imperatives [1,2]. The chemical oxygen demand (COD) is a parameter used widely to determine the amounts of organic pollutants in wastewater. The COD is defined as the number of oxygen equivalents consumed in the oxidation of organic compounds by strong oxidizing agents, such as dichromates and permanganates, and is indicative of the amount of organic pollutants present in the tested sample [3]. Conventional methods for determining the dichromate concentration suffer from several inherent drawbacks. For instance, they are time-consuming, exhibit low detection sensitivity, involve complex procedures, require the use of expensive (Ag_2_SO_4_) and toxic (Cr and Hg) chemicals, and result in the incomplete oxidation of the pollutants [1,4–11]. Consequently, secondary pollution is unavoidable when these methods are employed [12].

A number of alternative methods based on photocatalytic [13–15] or electrochemical [7,16–22] principles have been proposed and investigated. Although these methods have been shown to have a number of advantages over the traditional COD determination methods, they are far from perfect. The main drawback of processes based on the phenomenon of photocatalysis, such as those that employ discrete TiO_2_ particles and TiO_2_-coated nanofilms, is their low efficiency, owing to the ready recombination of the photogenerated electrons and holes in the particles and nanofilms [23].

In the case of electrochemical methods, numerous sensors, including copper electrodes [16], Cu/CuO electrodes [17], nano-PbO_2_-modified electrodes [18], F-doped PbO_2_-modified electrodes [19], RhO_3_/Ti electrodes [20], boron-doped diamond electrodes [7], rotating Pt ring–Pt/PbO_2_ disc electrodes [21], and cobalt oxide-modified glassy carbon electrodes [22] have been employed for determining the COD. The main merits of electrochemical COD methods are the following: a wide linear range, low cost, simplicity, short response time, and ease of automation. However, unstable background currents in the electrodes or the low oxidation capabilities of the chemicals involved, owing to which organic pollutants remain in the wastewater, lead to problems of poor reproducibility and accuracy [12].

We have developed a thermal biosensor for use in COD determination. The biosensor measures the amount of heat released during the oxidation of organic compounds in the test water samples [24–26]. The flow injection analysis (FIA) technique is normally used along with the assay [27]. Therefore, the amounts of the carrier solution and the test water sample can be readily controlled using this continuous analysis technique [28]. The height of the thermometric peak is proportional to the change in the enthalpy of the organic content in the test water sample [29,30]. In contrast to other devices used for COD measurements, the thermistor is insensitive to the optical, electrochemical, and other material properties of the test sample. Therefore, the degree of interference owing to the characteristics of the test sample is extremely low, and the obtained COD values are highly accurate [31]. In addition, the detection efficiency of the thermistor is very high.

In this study, we first investigated the feasibility of using Fenton's reagent, ozone, cerium sulfate solution, hydrogen peroxide, and periodic acid solution as oxidants for the determination of the COD using the thermistor. On the basis of the obtained results, periodic acid was chosen as the oxidant. Tri-distilled water was used as the carrier solution. Various experimental variables such as the flow rates of the mobile phases, the sample volume, the concentration and pH of the periodic acid solution, the alkali used for adjusting the pH, signal magnification, and the stability of the periodic acid solution were investigated with regard to their effects on the sensitivity and limit of detection of the analysis system. Glucose solutions of various concentrations were used to optimize the analysis parameters. A sample of tri-distilled water was used as the blank water sample. The experimental data were corrected using the values corresponding to the blank sample. The COD values detected using the FIA biosensor were correlated with those determined by the conventional manual method.

## 2. Materials and Methods

### 2.1. Reagents

All the chemicals used in the study were of analytical grade. Periodic acid, H_2_SO_4_ (98%), NaOH, NaCl, Na_2_HPO_4_, CH_3_COONa, glucose, K_2_Cr_2_O_7_, Ag_2_SO_4_, HgSO_4_, FeSO_4_·7H_2_O, C_12_H_8_N_2_·H_2_O, and (NH_4_)_2_Fe(SO_4_)_2_ were purchased from Sinopharm Group Chemical Reagent Co. Ltd., Beijing, China.

### 2.2. Apparatus

**Figure 1.**
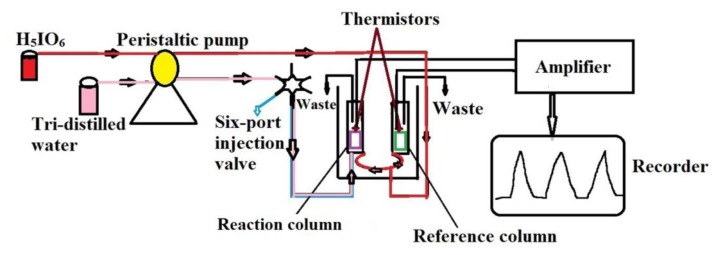
Schematic of the FIA biosensor.

The FIA biosensor used in this study has been described elsewhere previously [25]. A schematic of the device setup is shown in Figure 1.

The biosensor consists of a peristaltic pump with a tube mounted for each of its two channels, a six-port injection valve with a 350 μL sample loop, an amplifier, a computer, and two thermistors (Omik Bioscience AB, Lund, Sweden). The system employed in this study was an improved version of the conventionally used detector, which has only one outlet channel. The signals were recorded in the form of voltages. All the fluid samples used in the experiments were degassed to remove the air bubbles from the reaction column, as the bubbles can lead to an increase in signal noise. The pH values of all the solutions were detected using a pH Meter (Sartorius, Goettingen, Germany).

### 2.3. Glucose Solutions and Actual Water Samples

All the glucose solutions were prepared using tri-distilled water and were sonicated for 5 min before use. The working temperature was 30 °C, and the signal amplification factor was 100. A sample volume of 350 μL and a flow rate of 0.6 mL/min were used for all experiments, as well a 0.03 M solution of H_5_IO_6_, unless stated otherwise. The measurements were all optimized with respect to glucose. A stock solution of glucose with a concentration of 0.9375 g/L was prepared. Each sample was subjected to three parallel detections. The actual water samples were collected from different rivers, lakes and some drain outlets in Wuhan, including the Yangtze and Han rivers as well as Lake East, Lake Moon and filtered before testing.

### 2.4. Reference Method

The conventional dichromate method was used as the reference method to detect the COD values of the various samples, in keeping with the National Standards of China (GB 11914-89). This is the most commonly used COD method. H_2_SO_4_ (98%), glucose, K_2_Cr_2_O_7_, Ag_2_SO_4_, HgSO_4_, FeSO_4_·7H_2_O, C_12_H_8_N_2_·H_2_O, and (NH_4_)_2_Fe(SO_4_)_2_ were used. The obtained values were compared with those determined using the FIA biosensor.

## 3. Results and Discussion

Before using periodic acid solution in this study, we had investigated the feasibility of using Fenton's reagent, ozone, cerium sulfate solution, and hydrogen peroxide as oxidants for the determination of the COD using the thermal biosensor. These reagents, as opposed to periodic acid, generated excessive amounts of bubbles during glucose oxidation, leading to an increase in signal noise and an imbalance of the baseline. Therefore, we chose periodic acid solution as the most suitable oxidant.

### 3.1. Effect of Sample Volume

**Figure 2.**
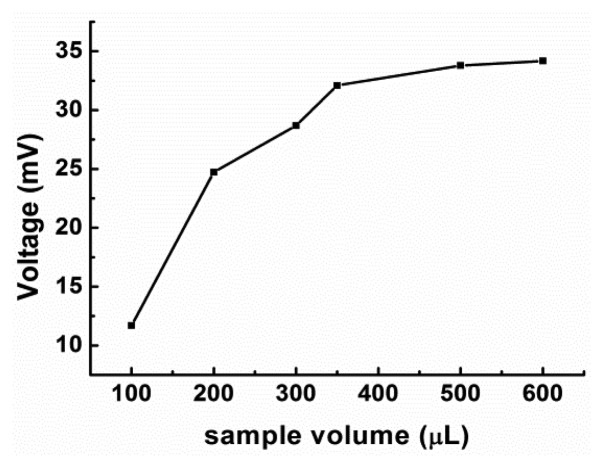
Effect of sample volume of a 93.75 mg/L glucose solution on the response of the FIA biosensor at a flow rate of 1.0 mL/min.

It is known that the sample volume [26,31,32] and flow rate [26,31,33] affect the sensitivity and linear range of the FIA biosensor assay. Although the sample volume for all the experiments was constant (350 μL), we wanted to elucidate its effects on the assay results. Therefore, measurements were performed with different volumes of 93.75 mg/L glucose solution and a 0.03 M periodic acid solution with a pH of 7; the flow rate was 1.0 mL/min. The results are shown in Figure 2.

On increasing the sample volume, the response signal also increased for volumes of up to 350 μL. For volumes greater than this, the signal remained steady. The differences in the response signal values corresponding to sample volumes of 350, 500, and 600 μL were not statistically significant. Hence, we used samples of 350 μL in the study.

### 3.2. Effect of Concentration of the Periodic Acid Solution

**Figure 3.**
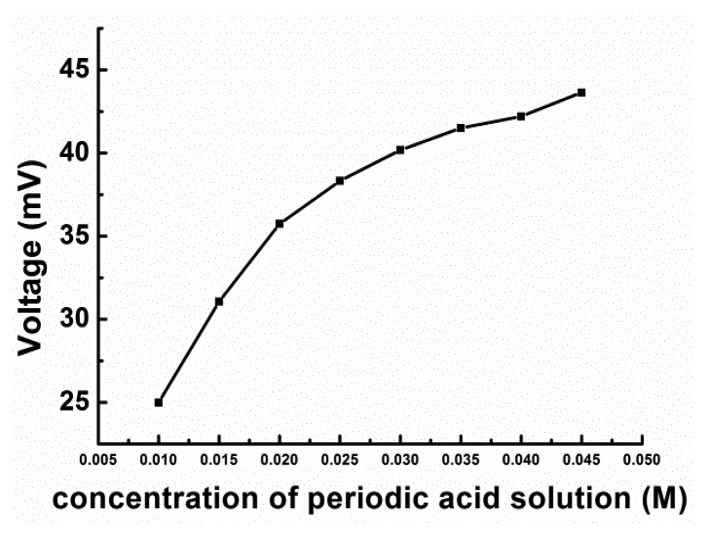
Effect of concentration of the periodic acid solution (pH 7) on the response of the FIA biosensor. A 350 μL sample of a 93.75 mg/L glucose solution was tested at a flow rate of 1.0 mL/min.

Experiments were performed to determine the effect of the concentration of the periodic acid solution on the assay results. A series of periodic acid solutions of varying concentrations (all having a pH of 7) were introduced into the biosensor, along with the 93.75 mg/L glucose solution, at the rate of 1.0 mL/min. As can be seen from Figure 3, the response signal increased rapidly for an increase in the concentration from 0.01 M to 0.025 M, but changed only slightly for further increases.

The signal was the steadiest for a concentration of 0.03 M; on the other hand, significant drift from the baseline was noticed when the concentration was greater than 0.03 M. H_5_IO_6_ solutions of high concentrations might damage the instruments owing to their high corrosivity. Therefore, on the basis of this set of results, an H_5_IO_6_ solution with a concentration of 0.03 M was employed for the assays.

### 3.3. Effect of Flow Rate

**Figure 4.**
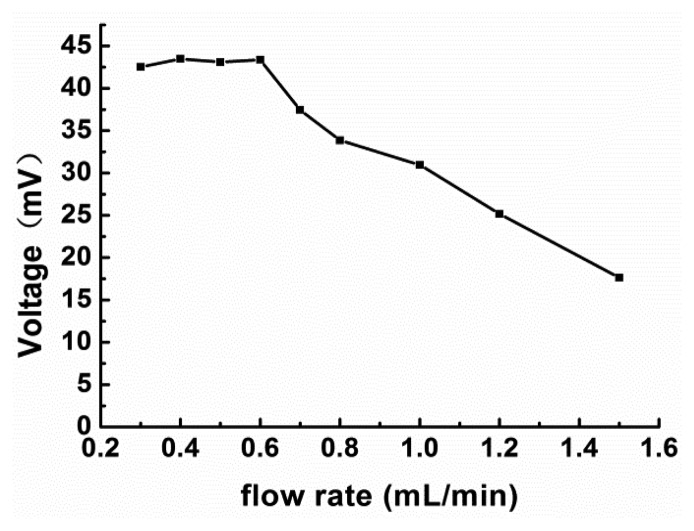
Effect of flow rate on the response of the FIA biosensor. A 350 μL sample of a 93.75 mg/L glucose solution was tested using a 0.03 M H_5_IO_6_ solution (pH 7).

Some studies have suggested that the flow rate has an effect on the response signal and that a suitable flow rate value is 0.5 [26,28] or 1.0 mL/min [24,31]. After investigating the effects of the flow rate, we found that a value of 0.6 mL/min was the most suitable for this study. The related data are displayed in Figure 4.

On increasing the flow rate, the response signal for the 93.75 mg/L glucose solution became smaller; however, the response time for each sample also decreased. The differences in the response signal values corresponding to flow rate values of 0.3, 0.4, 0.5, and 0.6 mL/min were not statistically significant, but the response time of 0.6 mL/min is the shortest. Taking into account the decrease in response time, a flow rate of 0.6 mL/min was employed during the assay experiments.

### 3.4. Effects of pH of the H_5_IO_6_ Solution and the Alkali Used to Change the pH

The results of preliminary experiments showed that the pH of the H_5_IO_6_ solution also impacted the assay results. An alkali was used to adjust the pH of the H_5_IO_6_ solution, and the most suitable alkali from among NaOH, Na_2_HPO_4_, and CH_3_COONa was chosen. The thermal response signals for a 937.5 mg/L glucose solution with a pH of 5 at a flow rate of 0.6 mL/min are listed in Table 1. The mean voltages corresponding to NaOH, Na_2_HPO_4_, and CH_3_COONa were 279.325, 325.156, and 233.644 mV, respectively. Thus, the responses in the cases of Na_2_HPO_4_ and CH_3_COONa were much higher than that obtained using NaOH. Moreover, when Na_2_HPO_4_ or CH_3_COONa was used to alter the pH, the H_5_IO_6_ solution grew turbid, and pH values of 8 and 10 could not be achieved. Therefore, we used NaOH to adjust the pH of the H_5_IO_6_ solution.

**Figure 5.**
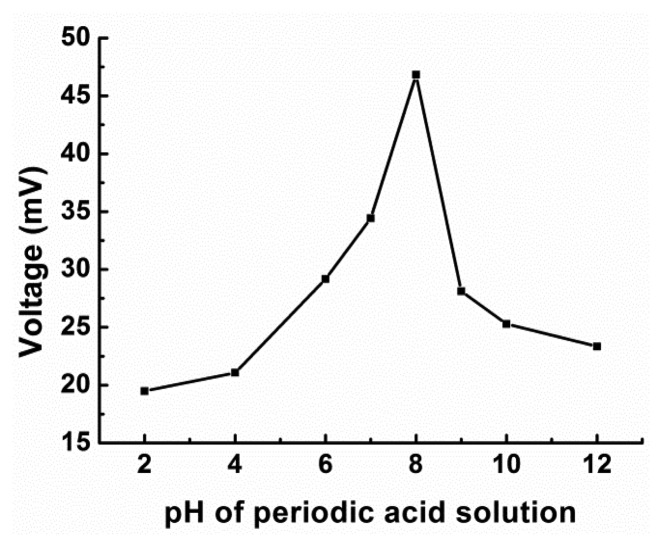
Effect of pH of H_5_IO_6_ on the response of the FIA biosensor. A 350 μL sample of a 93.75 mg/L glucose solution was tested using a 0.03 M H_5_IO_6_ solution at a flow rate of 0.6 mL/min.

The effect of the pH value of the H_5_IO_6_ solution on the assay was investigated using a 0.03 M H_5_IO_6_ solution and a 350 μL sample of a 93.75 mg/L glucose solution; the flow rate was 0.6 mL/min. As displayed in Figure 5, the response signal increased with an increase in the pH from 2 to 7, reaching its maximum value at pH 8. However, for further increases in the pH (*i.e.*, for increases from 8 to 12), the response signal declined markedly. The highest output was obtained at pH 8. It may be related to the nature of the instrument which can't endure strong acid or alkali and will be more stable in a milder environment. The result obtained in this study is consistent with 7.6 which was obtained by Weavers *et al.* [34] who used periodate for the reduction of the chemical oxygen demand in wastewater. Hence, a H_5_IO_6_ solution with a pH of 8 was used in all the experiments that follow.

### 3.5. Stability of H_5_IO_6_

**Figure 6.**
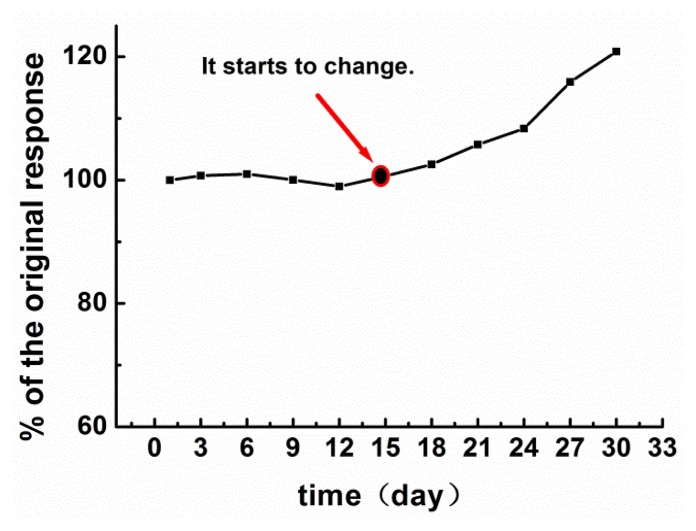
Stability of the response of the FIA biosensor with respect to a COD of 100 mg/L, as determined over 30 days.

The long-term stability of the oxidant during storage is one of the key factors influencing oxidant performance. We monitored the stability of H_5_IO_6_ over one month using a 93.75 mg/L glucose solution. The data, which were collected every three days, are displayed in Figure 6. It was found that the stability, which was defined as the percentage of the original response elicited, changed little from day 1 to day 15, but increased slightly between day 15 and day 30.

The differences in the stability values corresponding to days 1, 3, 6, 9, 12, and 15 were not statistically significant. Thus, it can be concluded that the stability of H_5_IO_6_ is very high. The increase in the response, that is, the increase in the degree of oxidation, after 15 days is likely owing to changes in H_5_IO_6_. H_5_IO_6_ can transform into HIO_4_ over time, as two water molecules are removed from the H_5_IO_6_ molecule. In addition, while H_5_IO_6_ is a weak acid, HIO_4_ is a strong one.

### 3.6. Reproducibility and Interference

The reproducibility of the responses obtained was investigated by measuring the voltages corresponding to a COD of 100 mg/L. The relative standard deviation (RSD) was 0.58% for a set of 11 values in a day and 4.48% for 72 samples in 20 days. The intraday and interday variations were 0.44% and 0.88%, respectively; both values were lower than 1%. Thus, the sensor exhibited highly reproducible behavior.

The most significant interference in the conventional dichromate method for the determination of COD is due to chloride ions [6,9,35–38]. On the one hand, they can reduce Cr(VI) to some extent and, on the other, precipitate with silver ions, thus removing the catalyst. Therefore, the effect of chloride ions on the biosensor's response was investigated. NaCl was used as the chloride source. In the presence of a 0.015 M chloride solution, the response corresponding to a COD of 100 mg/L remained unchanged, revealing that the sensor has a high tolerance level to chloride ions.

### 3.7. Calibration Curve

**Figure 7.**
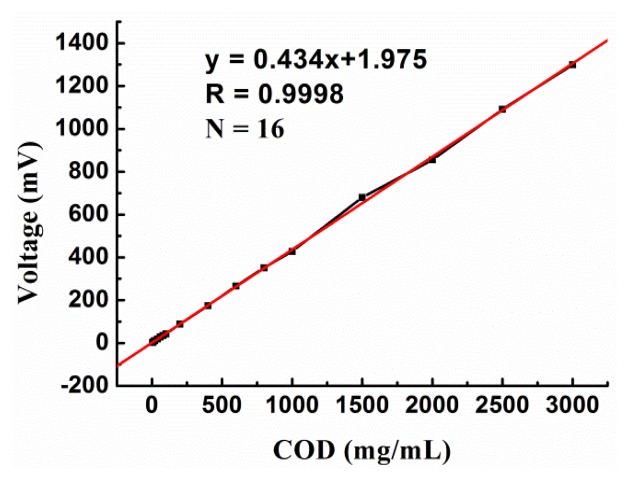
Calibration curve showing the linear range of the biosensor with respect to 350 μL glucose samples.

**Figure 8.**
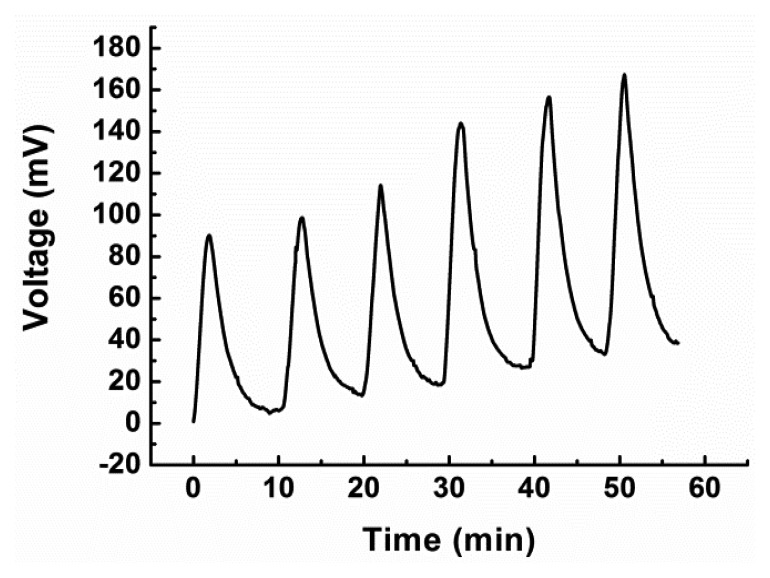
An actual sensor signal record for three determinations of 200 and 300 mg/L COD values.

The detection limit and linear range of the sensor were determined using glucose solutions of different concentrations. It was found that the thermal biosensor could be used for the determination of COD values ranging from 5 to 3000 mg/L; the regression coefficient was 0.9998 and the linear regression equation was Y (mV) = 0.434X (mg/L) + 1.975. For COD values higher than 3000 mg/L, the calibration curve remained flat. These results are presented in Figure 7. The detection limit of the sensor was 1.84 mg/L for a signal-to-noise ratio of 3. These values are sufficiently high to make the sensor suitable for the analysis of actual wastewater samples. An actual sensor signal record for three determinations of 200 and 300 mg/L COD values is presented in Figure 8.

### 3.8. Application Analysis of Actual Wastewater Samples

**Figure 9.**
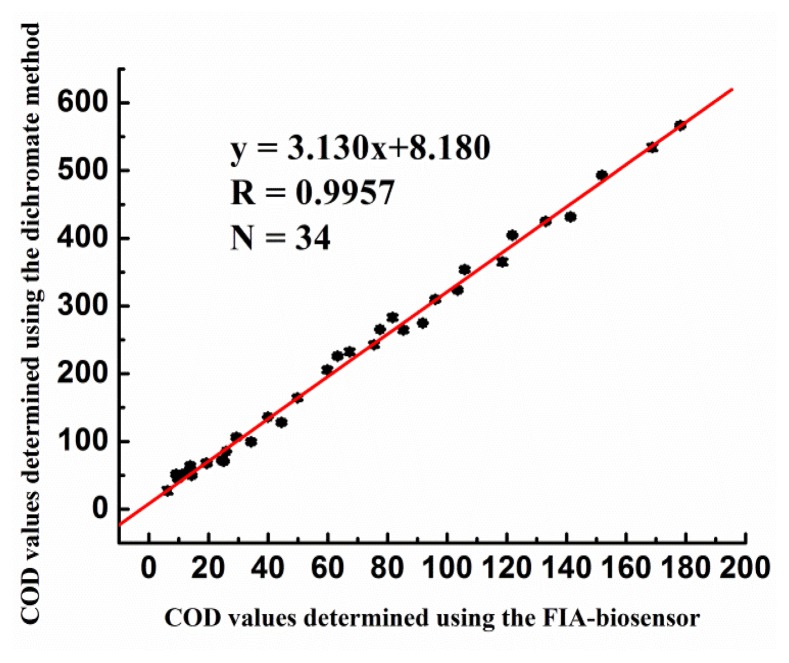
Graph showing the correlation between the COD values obtained using the FIA biosensor and those obtained using the dichromate method.

Any new measurement technique has to be compared with existing techniques, and the results obtained using the new technique must agree sufficiently well with those obtained using the conventional methods in order for the new technique to find wide application. Figure 9 shows the correlation between the results obtained using the dichromate method and those using the thermal biosensor; the values in the case of the thermal biosensor are shown on the *x*-axis, while those corresponding to the dichromate method are shown on the *y*-axis.

The number of samples used for the correlation experiments was 34. The regression coefficient for the values was 0.996. Periodic acid can only oxidize polysaccharides, which are incompletely oxidized into aldehydes. However, actual wastewater samples are likely to contain a variety of contaminants and not just polysaccharides. Dichromates can completely oxidize organic compounds. For these reasons, the COD values obtained using the dichromate method were approximately three times those obtained using the thermal biosensor.

## 4. Conclusions

The ability to sequentially inject more than 70 samples in 20 days while achieving highly reproducible results (RSD = 4.48%) attests to the robustness of the assay and the long-term stability of the sensor system. No detectable clogging was observed. The results obtained in this study indicate that this novel COD determination method, which is based on a thermal biosensor with a flow injection system, is a viable one. The sensitivity and repeatability of the assay are high enough to allow the sensor to be used in practical applications. The system is easy to operate, environmentally friendly, and highly stable. More importantly, it has a small response time (approximately 8 to 10 min). The assay system is thus suitable for the real-time monitoring of the COD values of water from rivers and lakes.

## Figures and Tables

**Table 1.** Effect of alkali used on the response of the biosensor.

**Alkali**	**ΔV/mV**		
Na_2_HPO_4_	288.820	265.540	283.614
CH_3_COONa	311.778	337.532	326.157
NaOH	230.390	240.252	230.285

## References

[1] Wang J., Li K., Zhang H., Wang Q., Wang Y., Yang C., Guo Q., Jia J. (2012). Condition optimization of amperometric determination of chemical oxygen demand using boron-doped diamond sensor. Res. Chem. Intermed..

[2] Shannon M.A., Bohn P.W., Elimelech M., Georgiadis J.G., Marinas B.J., Mayes A.M. (2008). Science and technology for water purification in the coming decades. Nature.

[3] Lee K.H., Ishikawa T., Sasaki S., Arikawa Y., Karube I. (1999). Chemical oxygen demand (cod) sensor using a stopped—Flow thin layer electrochemical cell. Electroanalysis.

[4] Jirka A.M., Carter M.J. (1975). Micro semiautomated analysis of surface and waste waters for chemical oxygen demand. Anal. Chem..

[5] Kim Y.-C., Lee K.-H., Sasaki S., Hashimoto K., Ikebukuro K., Karube I. (2000). Photocatalytic sensor for chemical oxygen demand determination based on oxygen electrode. Anal. Chem..

[6] Qu X., Tian M., Chen S., Liao B., Chen A. (2011). Determination of chemical oxygen demand based on novel photoelectro—Bifunctional electrodes. Electroanalysis.

[7] Yu H., Wang H., Quan X., Chen S., Zhang Y. (2007). Amperometric determination of chemical oxygen demand using boron-doped diamond (bdd) sensor. Electrochem. Commun..

[8] Zhang A., Zhou M., Han L., Zhou Q. (2010). Amperometric determination of chemical oxygen demand via the functional combination of three digestion types. Electroanalysis.

[9] Zhang A., Zhou M., Zhou Q. (2011). A combined photocatalytic determination system for chemical oxygen demand with a highly oxidative reagent. Anal. Chim. Acta.

[10] Baton D., Glescer L., Greenberg A. (1995). Standard Methods for Examination of Water and Waste Water.

[11] Domini C.E., Hidalgo M., Marken F., Canals A. (2006). Comparison of three optimized digestion methods for rapid determination of chemical oxygen demand: Closed microwaves, open microwaves and ultrasound irradiation. Anal. Chim. Acta.

[12] Han Y., Qiu J., Miao Y., Han J., Zhang S., Zhang H., Zhao H. (2011). Robust TiO_2_/BDD heterojunction photoanodes for determination of chemical oxygen demand in wastewaters. Anal. Methods.

[13] Kim Y.-C., Sasaki S., Yano K., Ikebukuro K., Hashimoto K., Karube I. (2001). Photocatalytic sensor for the determination of chemical oxygen demand using flow injection analysis. Anal. Chim. Acta.

[14] Kim Y.-C., Sasaki S., Yano K., Ikebukuro K., Hashimoto K., Karube I. (2002). A flow method with photocatalytic oxidation of dissolved organic matter using a solid-phase (TiO_2_) reactor followed by amperometric detection of consumed oxygen. Anal. Chem..

[15] Dan D., Sandford R.C., Worsfold P.J. (2005). Determination of chemical oxygen demand in fresh waters using flow injection with on-line UV-photocatalytic oxidation and spectrophotometric detection. Analyst.

[16] Lee K.-H., Ishikawa T., McNiven S., Nomura Y., Hiratsuka A., Sasaki S., Arikawa Y., Karube I. (1999). Evaluation of chemical oxygen demand (COD) based on coulometric determination of electrochemical oxygen demand (EOD) using a surface oxidized copper electrode. Anal. Chim. Acta.

[17] Silva C.R., Conceição C.D., Bonifácio V.G., Fatibello Filho O., Teixeira M.F. (2009). Determination of the chemical oxygen demand (COD) using a copper electrode: A clean alternative method. J. Solid State Electrochem..

[18] Ai S., Gao M., Yang Y., Li J., Jin L. (2004). Electrocatalytic sensor for the determination of chemical oxygen demand using a lead dioxide modified electrode. Electroanalysis.

[19] Li J., Li L., Zheng L., Xian Y., Ai S., Jin L. (2005). Amperometric determination of chemical oxygen demand with flow injection analysis using F-PbO_2_ modified electrode. Anal. Chim. Acta.

[20] Li J., Li L., Zheng L., Xian Y., Jin L. (2006). Rh_2_O_3_/Ti electrode preparation using laser anneal and its application to the determination of chemical oxygen demand. Meas. Sci. Technol..

[21] Westbroek P., Temmerman E. (2001). In line measurement of chemical oxygen demand by means of multipulse amperometry at a rotating Pt ring—Pt/PbO_2_ disc electrode. Anal. Chim. Acta.

[22] Wang J., Wu C., Wu K., Cheng Q., Zhou Y. (2012). Electrochemical sensing chemical oxygen demand based on the catalytic activity of cobalt oxide film. Anal. Chim. Acta.

[23] Wang J., He Y., Tao J., He J., Zhang W., Niu S., Yan Z. (2010). Enhanced photodegradation of dyes on titania-based photocatalysts by adding commercial GeO_2_ in aqueous suspension. Chem. Commun..

[24] Xie B., Ramanathan K., Danielsson B. (2000). Mini/micro thermal biosensors and other related devices for biochemical/clinical analysis and monitoring. TrAC Trends Anal. Chem..

[25] Xie B., Danielsson B. (2007). Thermal biosensor and microbiosensor techniques. Handbook of Biosensors and Biochips.

[26] Chen Q., Andersson A., Mecklenburg M., Xie B. (2013). Fast determination of antibiotics in whole blood. Clin. Microbiol. Infect..

[27] Decristoforo G., Danielsson B. (1984). Flow injection analysis with enzyme thermistor detector for automated determination of .beta.-lactams. Anal. Chem..

[28] Mishra G.K., Mishra R.K., Bhand S. (2010). Flow injection analysis biosensor for urea analysis in adulterated milk using enzyme thermistor. Biosens. Bioelectron..

[29] Ramanathan K., Danielsson B. (2001). Principles and applications of thermal biosensors. Biosens. Bioelectron..

[30] Lerchner J., Wolf A., Wolf G., Baier V., Kessler E., Nietzsch M., Krügel M. (2006). A new micro-fluid chip calorimeter for biochemical applications. Thermochim. Acta.

[31] Zheng Y.-H., Hua T.-C., Xu F. (2005). A novel thermal biosensor based on enzyme reaction for pesticides measurement. J. Environ. Sci..

[32] Yerian T.D., Christian G.D., Ruzicka J. (1988). Flow injection analysis as a diagnostic tool for development and testing of a penicillin sensor. Anal. Chem..

[33] Xie B., Mecklenburg M., Danielsson B., Öhman O., Norlin P., Winquist F. (1995). Development of an integrated thermal biosensor for the simultaneous determination of multiple analytes. Analyst.

[34] Weavers L.K., Hua I., Hoffmann M.R. (1997). Degradation of triethanolamine and chemical oxygen demand reduction in wastewater by photoactivated periodate. Water Environ. Res..

[35] Cuesta A., Todolí J.L., Mora J., Canals A. (1998). Rapid determination of chemical oxygen demand by a semi-automated method based on microwave sample digestion, chromium (vi) organic solvent extraction and flame atomic absorption spectrometry. Anal. Chim. Acta.

[36] Moore W.A., Kroner R.C., Ruchhoft C. (1949). Dichromate reflux method for determination of oxygen consumed. Anal. Chem..

[37] Su Y., Li X., Chen H., Lv Y., Hou X. (2007). Rapid, sensitive and on-line measurement of chemical oxygen demand by novel optical method based on uv photolysis and chemiluminescence. Microchem. J..

[38] Zhang S., Li L., Zhao H., Li G. (2009). A portable miniature uv-led-based photoelectrochemical system for determination of chemical oxygen demand in wastewater. Sens. Actuators B Chem..

